# Keratomalacia and corneal perforation in vitamin A deficiency: Anterior-segment optical-coherence-tomography and histological findings

**DOI:** 10.1016/j.ajoc.2024.102169

**Published:** 2024-09-10

**Authors:** Julia M. Weller, Theofilos Tourtas, Friedrich E. Kruse

**Affiliations:** Department of Ophthalmology, Friedrich-Alexander University Erlangen-Nürnberg, Erlangen, Germany

**Keywords:** Vitamin A deficiency, Corneal perforation, Optical coherence tomography

## Abstract

**Purpose:**

To describe the histological findings and results of anterior-segment optical-coherence-tomography (AS-OCT) in patients with severe keratomalacia and corneal perforation due to vitamin-A deficiency (VAD).

**Observations:**

Four patients (3 female, 1 male) with moderate to severe VAD were included in this single-center case series. Keratomalacia/corneal perforation was diagnosed by slit-lamp examination. The findings were documented using photographs and AS-OCT imaging (CASIA-2, *Tomey corporation, Nagoya, Japan).* Ocular and general medical findings including causes of VAD are reported. VAD was severe (<20 ng/ml) in two patients with chronic alcoholism, and moderate (205/231 ng/ml) in two patients with cachexia. Corneal perforation occurred in 3 out of 4 patients. One patient had severe keratomalacia with impending perforation and massive conjunctival folds with Bitot's spots, which vanished soon after initiation of vitamin A supplementation. In three cases, corneal specimens obtained during anterior lamellar keratoplasty were assessed histologically by light-microcopy. The histological specimens showed massive epithelial thickening and a pronounced granular layer of the corneal epithelium.

**Conclusions and importance:**

Keratomalacia leading to corneal perforation is a rare, but possible complication also in countries with sufficient general food supply. The clinico-pathological correlation between AS-OCT and histological findings in patients with VAD is shown in this case series. Typical Bitot's spots were present only in one patient and can be visualized by AS-OCT.

## 1 Introduction

The fat-soluble vitamin A can be ingested from animal food sources (meat, eggs, dairy products etc.) or from plant sources (dark-green vegetables, carrots etc.) as provitamin A. In the liver, the metabolism from provitamin A to retinol takes place in a zinc-dependent matter.^1^

Worldwide, primary vitamin A deficiency (VAD) is still one of the most important public health problems due to malnutrition in lower-income countries.^2^ In 2009, about one third of preschool children worldwide had reduced serum retinol levels, with signs of night blindness in 0.9 % of the children. By strategic vitamin A supplementation, a reduction of the rates of clinical (Bitot's spots) and subclinical VAD has been achieved for example in preschool children in Ethiopia.^3^ Nevertheless, it is still one of the leading causes of night blindness and corneal disease in undernourished children.

VAD is a rare condition in countries with sufficient general food supply, but may occur in patients with malnutrition due to chronic alcoholism, or malabsorption due to gastrointestinal diseases, for example celiac disease, Crohn's disease, primary biliary cirrhosis, chronic liver diseases, severely restricted diets, anorexia, or short bowel syndrome.^4^, ^5^, ^6^, ^7^, ^8^, ^9^, ^10^

Vitamin A/retinoic acid is necessary for regeneration of the corneal epithelium, goblet cell differentiation, and rhodopsin metabolism.^11^^,^^12^ Ocular findings in VAD regard the anterior segment as well as the posterior segment: in the conjunctiva, Bitot's spots are a typical finding, which is characterized by keratinization of the conjunctiva in the bulbar conjunctiva at 3 and 9 o'clock with a foamy appearance and is reversible after vitamin A supplementation. Furthermore, severe dry eye disease is typical for VAD contributing to corneal ulcerations in some cases.^4^, ^5^, ^6^, ^7^, ^8^, ^9^^,^^13^ Several reports have been published about corneal perforation in patients with VAD in countries with sufficient general food supply.^5^, ^6^, ^7^, ^8^, ^9^, ^10^

In the retina, hypovitaminosis A leads to reduced light sensitivity with night blindness/nyctalopia. In contrast to retinal dystrophies, night blindness/nyctalopia in VAD is reversible after supplementation of vitamin A.

The purpose of this study is to show the histological and anterior-segment optical coherence tomography (AS-OCT) findings in four patients with corneal affection and perforation in the setting of VAD.

## 2 Patients and methods

### 2.1 Patients

In this retrospective, single-center study, the medical records at a tertiary referral center for Ophthalmology (Department of Ophthalmology, Friedrich-Alexander-University Erlangen-Nürnberg (FAU), Erlangen, Germany) were screened for cases with proven VAD and ocular morbidity. Four patients fulfilled these criteria. Written informed consent was obtained from all patients included in this study. The institutional review board waived the need for approval of this study.

Main study parameters were reason for consultation, age of patient at presentation, sex, best corrected visual acuity, cause of VAD, ocular findings at the anterior segment, clinical course, treatment modalities, and follow-up time.

### 2.2 Methods

Best corrected visual acuity (BCVA) was measured using standard number optotypes in a clinical setting. Slit lamp examination was performed in all patients with fluorescein staining of the surface. Dependent on the slit lamp findings, further measurements (ultrasound, optical coherence tomography, etc.) were initiated. Photographs of the anterior segment were taken at first presentation and during the follow-up. Anterior-segment optical coherence tomography (AS-OCT) measurements of the cornea were taken with the device CASIA-2 (*Tomey corporation, Nagoya, Japan*).

### 2.3 Histological analysis

The corneal material excised during lamellar keratoplasty of three patients (cases #1, 2, and 4) were fixed in buffered 10 % formaldehyde solution (pH 7.2), dehydrated, and embedded in paraffin. Serial sections cut at 5 μm were stained with hematoxylin and eosin (HE) and periodic acid-Schiff (PAS).

## 3 Results

The results of case #1–4 are summarized in Table 1.

**Table 1:** – Summary of patients’ characteristics, general medical findings, ocular findings, laboratory results, and treatment.

	Case 1	Case 2	Case 3	Case 4
Age at presentation	70 y	35 y	65 y	72 y
Sex	male	female	female	female
BCVA at presentation	OD: 20/630 OS: light perception	OD: 20/25 OS 20/200	OD: no light perception OS: 40/200	OD: 20/500 (advanced glaucoma) OS: 20/80
Cause for vitamin A deficiency	Suspected alcoholism, reduced general health state	Alcoholism	Cachexia (body mass index: 15.8 kg/m^2^)	Liver cirrhosis, cachexia (body mass index 19.5 kg/m^2^)
Laterality of eye affection	bilateral	unilateral	bilateral	unilateral
General history	Liver fibrosis with beginning cirrhosis, suspected alcoholism, nicotine addiction, psoriasis	Liver cirrhosis, ascites, alcoholism, asthma	Cachexia of unknown reason	Liver cirrhosis, anemia, chronic obstructive pulmonary disease, rheumatoid arthritis, Graves' disease, gastric ulcer, reflux esophagitis, chronic pancreatitis, osteoporosis, cardial decompensation, tricuspid valve insufficiency
Conjunctival alterations (Bitot's spots)	Bitot's spots, keratinized conjunctiva	No Bitot's spots	Mild conjunctival injection, no Bitot's spots, keratinization of the conjunctivalized cornea	Conjunctival hyperemia, no Bitot's spots
Corneal findings	OD: massive staining, focal infiltrates, erosion, corneal edema, Descemet folds OS: bulging of the inferior corneal hemisphere, keratomalacia, vascularizations, flattened anterior chamber	OD: normal OS: peripheral perforated corneal ulcer with iris prolaps	OD: scarred cornea, athalamia OS: superficial corneal ulcer, infectious nfiltrate	OD: normal OS: paracentral focal white stromal infiltrate with overlying erosion; stromal thinning
Corneal sensitivity (esthesiometry)	n/a	6/6	4/6	3/6
Microbiology	Negative	Negative	Negative	Positive for *Candida albicans* (first sample), positive for *Stenotrophomonas maltophilia* (second sample after 1 month)
Ocular diagnosis	OD: keratitis due to massive corneal xerosis OS: keratomalacia with large ulceration and retrocorneal fibrin	OD: without pathological findings OS: perforated corneal ulcer	OD: blindness due to assumed previous corneal perforation OS: superficial infectious corneal ulcer	OD: without pathological findings OS: mycotic corneal ulcer due to exposition keratopathy and vitamin A deficiency, followed by bacterial superinfection and perforation
Treatment	OD: topical antibiotics (cefuroxim, tobramycin) and lubricants OS: anterior lamellar tectonic keratoplasty with amniotic membrane patch	OU: lubricants OS: anterior lamellar tectonic keratoplasty	OD: lubricants, no surgery due to poor prognosis OS: antibiotic eye drops, lubricants, topical steroids	OD: lubricants OS: topcial antimycotics hourly, followed by antibiotics due to bacterial superinfection; finally anterior lamellar keratoplasty with lateral tarsorraphy due to corneal perforation
Vitamin A level (normal range: 300–800 ng/ml)	<20 ng/ml	<20 ng/ml	205 ng/ml	231 ng/ml
Laboratory*	hypoproteinemia (48.8 g/l), hypalbuminemia (30.5 g/l) low vitamin B1 (25 μg/l) slightly reduced vitamin B12 (203 pg/ml) low 25-OH-vitamin-D3 (4.3 ng/ml) low transferrin (1.24 g/l)	high MCH (37 pg) high MCV (112 fl) high CRP (40.7 mg/l) high LDH (397 U/l) high gamma-GT (184 U/l) high white blood cells (12.300/μl)	high MCH high MCV gamma GT normal ALT/AST normal	hypoproteinemia (59 g/l) high gamma GT (77 U/l) high AST (60 U/l) high ALT (49 U/l) low platelets (94.000/μl) low hemoglobin (10.8 g/dl) high α-amylase (141 U/l) high lipase (219 U/l)
Follow-up time	–	15 months	2 months	2 months
Ocular co-factors	–	–	Blepharitis posterior	Exposition keratopathy

*.

alpha-amylase (normal <110 U/l).

ALT = alanine transaminase (normal <35 U/l).

AST = aspartate transaminase (normal <35 U/l).

CRP = C-reactive protein (normal <5 mg/l).

gamma-GT = gamma-glutamyltransferase (normal <40 U/l).

hemoglobin (normal 12–15.5 g/dl).

LDH = lactat dehydrogenase (normal <250 U/l).

lipase (normal <60 U/l).

MCH = mean corpuscular hemoglobin (normal 27–32 pg).

MCV = mean corpuscular volume (normal 83–98 fl).

serum protein (normal 60–68 g/l).

serum albumin (normal 35–55 g/l.

transferrin (normal 2.0–3.6 g/l.

25-OH-vitamin-D3 (normal: 30–70).

### 3.1 Case #1

**Fig. 1:**
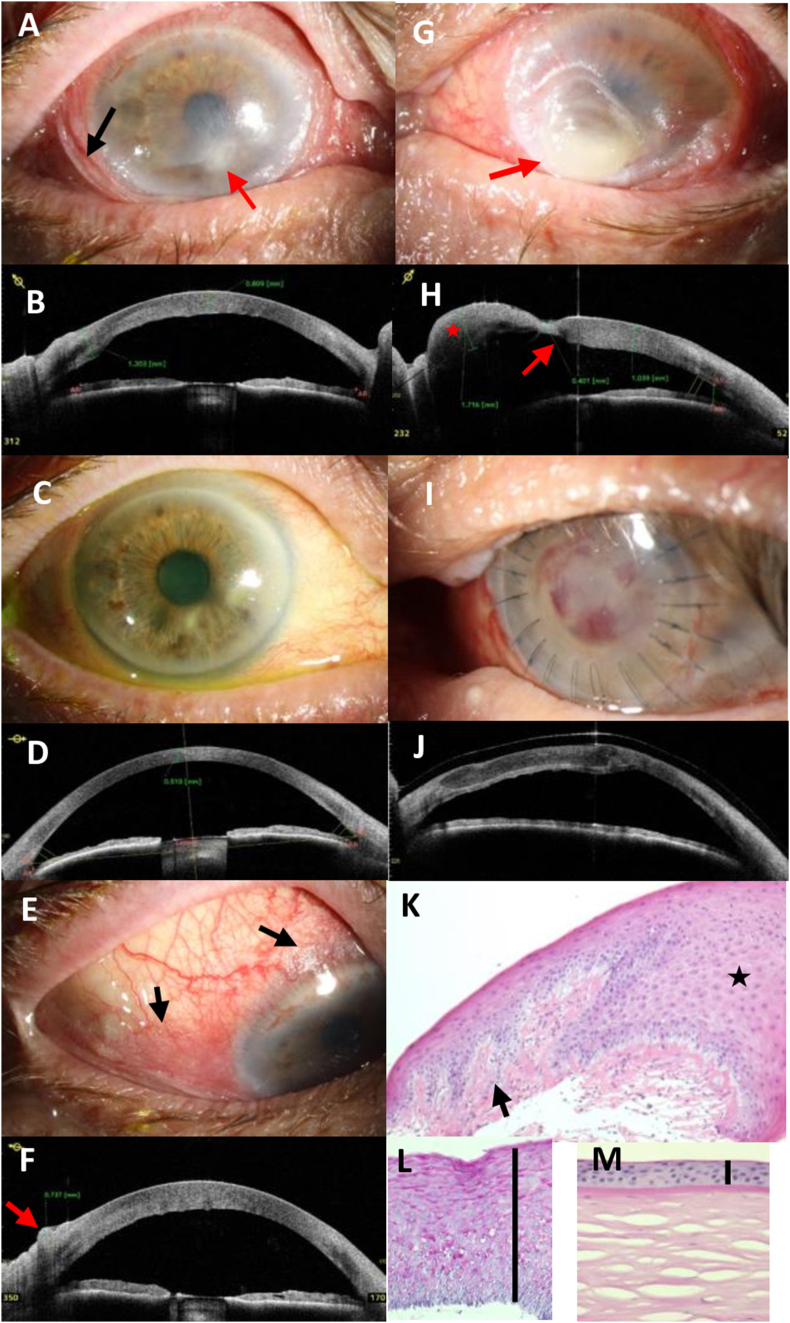
Case #1: clinical photos and AS-OCT imaging of a 70-year-old male patient with severe vitamin A deficiency due to chronic liver disease. A) Photograph, OD, at first presentation: a paracentral stromal white infiltrate (red arrow), massive Bitot's spots in the bulbar conjunctiva (black arrow), Descemet folds, and stromal thickening. B) AS-OCT, OD, at first presentation: thickening of the bulbar conjunctiva, edema of the corneal stroma. C) Photograph, OD, after three weeks: clearance of the cornea with focal stromal scar, and a smoother bulbar conjunctiva without hyperemia. D) AS-OCT, OD, after three weeks: normal corneal thickness. E) Photograph, OD, at first presentation showing Bitot's spots (arrows) in the temporal and superior bulbar conjunctiva. F) AS-OCT, OD, at first presentation, Bitot's spots can be detected as wavy folding of the bulbar conjunctiva overlapping the peripheral cornea with thickening of the conjunctiva up to 737 μm (arrow). G) Photograph, OS, at first presentation: massive conjunctival folds and keratinization (Bitot's spots), keratomalacia with with-yellow bulging of the stroma (arrow). H) AS-OCT, OS, at first presentation: bulging of the inferior hemisphere of the cornea (asterisk), focal stromal thinning to 401 μm (arrow) beside stromal edema (1039 μm). I) Photograph, OS, after three weeks: anterior lamellar corneal graft with single sutures, slight hemorrhage in the interface J) AS-OCT, OS, after three weeks: good adaptation of the lamellar graft, normal anatomy of the anterior chamber K) Histology, OS: thickened corneal epithelium (asterisk) with focal papillomatosis (arrow), loss of Bowman's layer, adherent irregular stromal lamellae (HE, magnification 50×) L) Histology, OS: excessive thickening of the epithelium to about 20 cell layers with PAS-positive granular material in the apical layers and slight keratinization (PAS, 100×, the bar indicates the epithelial thickness) M) Histology, control eye: normal corneal epithelium (about 5 cell layers) with underlying Bowman's layer and stroma for comparison (PAS, 100×, the bar indicates the epithelial thickness).

A 70-year-old male patient presented with bilateral painless loss of vision and epiphora of the eyes which had started 2 weeks ago. He was in a reduced general state of health, with normal body weight (body mass index 21.6 kg/m^2^). According to his relatives, he usually avoided contact to the health system. His general medical history was positive for psoriasis, chronic alcohol consumption (about 1 L of beer per day), and cigarette smoking. Visual acuity was reduced to 20/630 (OD) and light perception (OS). The cornea of the right eye had several small stromal infiltrates, epithelial defects, stromal edema and Descemet folds (Fig. 1 A/B). In the left eye, the cornea was cloudy due to a large ulcer (measuring 5.8 × 6.7 mm) with a diffuse yellowish infiltrate, retrocorneal fibrin and bulging of the inferior corneal hemisphere (Fig. 1 G/H). Besides, stromal vascularizations and edema with Descemet folds in the upper half of the cornea were apparent. The bulbar conjunctiva of both eyes showed marked Bitot's spots with glistening of the keratinized surface (Fig. 1 E/F).

AS-OCT was used to measure the thickness of the cornea (right eye: 809 μm) and the conjunctiva and to visualize the bulging of the left cornea due to advanced keratomalacia (Fig. 1 B/F/H).

Laboratory tests revealed low serum levels of albumin, general protein, vitamin A (<20 ng/ml, normal range: 300–800 ng/ml), vitamin B1 (25 μg/l) vitamin B12 (133 pg/ml), and vitamin D3 (4.3 ng/ml). In abdominal ultrasound, fibrosis with beginning cirrhosis of the liver was diagnosed. Supplementation of vitamin B1, B12, D3 and vitamin A was started orally (200.000 I.E. per day orally for 2 days).

The right eye was treated with topical antibiotics and lubricants leading to fast clearance of the stroma, reduction of stromal thickness, and normalization of the conjunctival appearance (Fig. 1 C/D). In the left eye, an anterior tectonic lamellar keratoplasty with amniotic membrane patch was performed without complications (Fig. 1 I/J).

By AS-OCT the morphological alterations after successful treatment of VAD were monitored and the correct alignment of the lamellar corneal graft in the stromal bed was documented (Fig. 1 D/J). The corneal tissue (anterior stromal lamella), which was removed at preparation of the lamellar stromal bed during keratoplasty, was obtained and sent for histological examination.

### 3.2 Case #2

A 35-year-old female was referred with a history of ocular irritation for three weeks in her left eye. The medical history of the patient revealed severe liver cirrhosis with ascites due to chronic alcoholism.

**Fig. 2:**
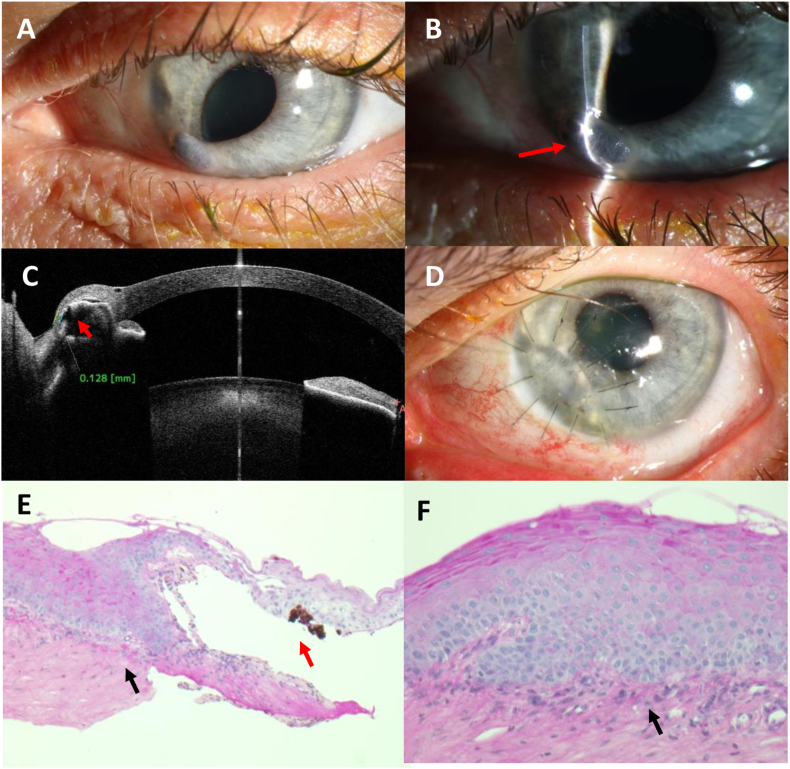
Case #2: clinical photos and AS-OCT imaging of a 35-year-old female with severe vitamin A deficiency due to chronic liver disease and alcoholism A) Overview photograph, OS, at first presentation: distortion of the pupil and prolapse of the iris at 7 o'clock without visible signs of inflammation. B) Slit-lamp photograph, OS, at first presentation: prolapse of the iris through a perforated corneal ulcer (arrow). C) AS-OCT, OS, at first presentation: thinning of the corneal stroma at the periphery with incarceration of the iris in the stroma (arrow). D) Photograph, OS, after two months: well-adapted, fusiform, anterior lamellar tectonic graft without signs of inflammation. E) Histology, OS: thickened corneal epithelium covering the irregular stromal collagen; loss of Bowman's layer (black arrow); steep rim of the corneal ulcer; overlying keratinizing epithelium with adherent iris pigment (red arrow) due to corneal perforation (PAS, 50×) F) Histology, OS: massively thickened epithelium as in case #1; slight papillomatosis; absence of Bowman's layer (black arrow) (PAS, 100×).

At 8 o'clock at the corneal periphery of the left eye, a perforated corneal ulcer with iris prolapse was seen at the slit lamp (Fig. 2 A/B). AS-OCT was used to visualize the perforation of the peripheral cornea with incarceration of the iris (Fig. 2 C). There were no signs of Bitot's spots, no infection, no hypopyon, no vitreous cells. The right eye appeared normal with visual acuity of 20/25.

Laboratory tests revealed a severely reduced vitamin A serum level (<20 ng/ml). A tectonic anterior lamellar keratoplasty was performed (Fig. 2 D), and the removed corneal tissue was examined histologically (Fig. 2 E/F). The corneal graft healed well and the patient was dismissed. Further medical work-up of her liver dysfunction was initiated and a supplementation of vitamin A orally was started.

At the follow-up visits 12 and 15 months after first presentation, the corneal sutures were removed. The corneal graft showed slight vascularizations and both eyes had 1+ corneal staining. Treatment consisted of lubricants including vitamin A ointment (both eyes), topical steroid eye drops once daily (OS), and systemic vitamin A supplementation.

### 3.3 Case #3

A 65-year-old female patient presented with foreign body sensation and slight pain in her left eye. The right eye had been blind for several years for unknown reason; the patient had not visited an ophthalmologist in this regard. The patient had severe underweight (body mass index 15.8 kg/m^2^) without known bowel disease. She reported that she usually eats two meals per day (no special diet or allergies), drinks rarely alcohol, and negated drug consumption.

**Fig. 3:**
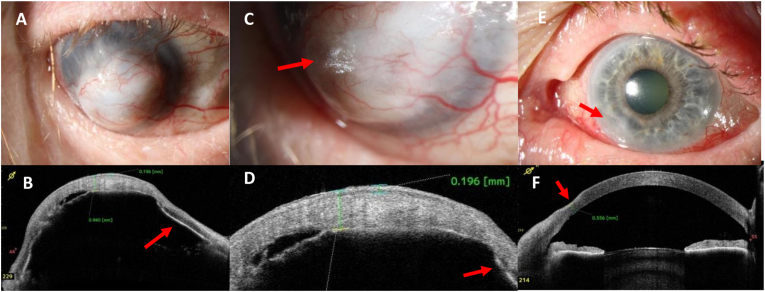
Case #3: clinical photos and AS-OCT imaging of a 65-year-old female with cachexia A) Photograph, OD, at first presentation: bulging of the whitish cornea, which is completely covered by conjunctival tissue; athalamia. B) AS-OCT, OD, at first presentation: the cornea is thickened to 940 μm at the center and bulges forward due to keratomalacia; focal cleft between stroma and Descemet's membrane; complete attachment of the iris to the cornea (arrow). C) Photograph, OD, higher magnification: keratinization of the corneal surface similar to conjunctival Bitot's spots (arrow). D) AS-OCT, OD, higher magnification: massive thickening of the epithelium to 196 μm, attached iris to the cornea (arrow). E) Photograph, OS, at first presentation: hyperemia of the bulbar conjunctiva, hazy lesion at 7–8 o'clock peripherally (arrow). F) AS-OCT, OS, at first presentation: slight corneal thinning (556 μm) at 7–8 o'clock (arrow).

Visual acuity of the right eye was no light perception and a diffuse vascularized scar of the cornea was seen. Because of complete athalamia, previous corneal perforation was suspected (Fig. 3 A/B). AS-OCT revealed bulging of the cornea as in keratectasia (Fig. 3 B). The epithelium could be demarcated as superficial, thickened layer (196 μm) and the iris was shown to be completely attached to the posterior surface of the cornea (Fig. 3 B/D).

In the left eye, BCVA was 40/200, corneal sensitivity was slightly reduced, and posterior blepharitis with conjunctival injection was noticed. In the left eye, there was a peripheral corneal ulcer (2.0 × 1.4 mm) with underlying stromal infiltrate and stromal thinning to 556 μm at the periphery, and anterior chamber cells (Fig. 3 E/F).

Because of the low body mass index, malnutrition was suspected and vitamin A serum levels were measured, revealing a vitamin A level of 205 ng/ml. Further gastrointestinal examination and vitamin A supplementation was initiated. Under treatment of the left eye with topical antibiotics (cefuroxim/tobramycin eye drops hourly, vitamin A ointment 5 times a day), the superficial corneal ulcer healed within 6 days.

### 3.4 Case #4

**Fig. 4:**
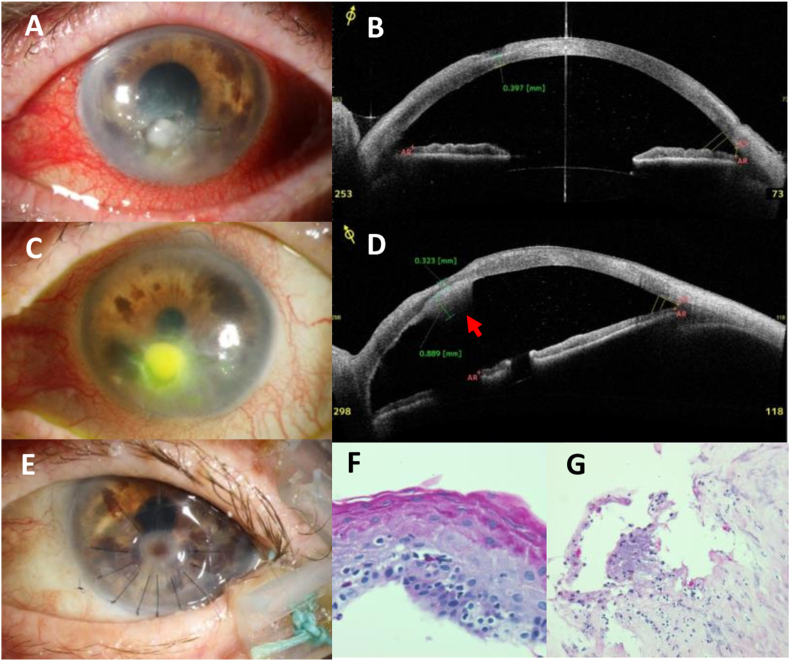
– Case #4: clinical photos, AS-OCT imaging, and histological findings of a 72-year-old multimorbid female A) Photograph, OS, at first presentation: hyperemia of the bulbar conjunctiva, focal white corneal infiltrate with overlying erosion; surrounded by irregular epithelium. B) AS-OCT, OS, at first presentation: focal corneal thinning to 397 μm C) Photograph, OS, after 1 month: enlarged infiltrate/retrocorneal fibrin deposit; persisting corneal erosion D) AS-OCT, OS, after 1 month: progressive corneal thinning (323 μm); retrocorneal attached fibrin (889 μm, arrow). E) Photograph, OS, after surgery: anterior lamellar tectonic keratoplasty and lateral tarsorrhaphy has been performed for the repair of the focally perforated corneal ulcer. F) Histology, periodic acid-Schiff/PAS stain, 200 ×: thickening of the corneal epithelium, PAS-positive granular deposits in the superficial corneal layers. G) Histology, PAS stain, 100 ×: irregular, partially necrotic corneal stromal layers with focal PAS-positive material (presumed non-vital fungal elements), few inflammatory cells.

A 72-year-old female presented with a paracentral corneal ulcer with a white stromal infiltrate and thinning of the cornea to 397 μm in her left eye (Fig. 4 A/B). Her medical history was positive for many diseases, most notably liver cirrhosis, gastric ulcer, chronic obstructive pulmonary disease (COPD), chronic pancreatitis, and cachexia. Her husband reported that the eyelids did not close completely at night. Laboratory tests showed a reduced serum vitamin A level (231 ng/ml) and increased liver enzymes.

Corneal scraping revealed *Candida albicans* and antimycotic topical treatment with amphotericin B/voriconazole eye drops hourly was initiated. The infiltrate diminished slowly, but the corneal epithelial wound healed very slowly. After 1 month, there was a new, enlarging stromal infiltrate with retrocorneal attachment of fibrin, and further stromal thinning (Fig. 4 C/D). *Stenotrophomonas maltophilia* was detected by microbiological culture, and the treatment regimen was changed to ofloxacin eye drops hourly. After stabilization of the infection, an amniotic membrane patch was performed for the treatment of persistent corneal erosion. Nevertheless, the cornea perforated within 6 days, and the decision was made for tectonic anterior lamellar keratoplasty with lateral tarsorrhaphy (Fig. 4 E). The graft integration and wound healing was well, but the general condition of the patient deteriorated due to anemia, making erythrocyte transfusion necessary. In this case, AS-OCT helped to measure the residual stromal thickness and to differentiate between the stromal infiltrate and the retrocorneal fibrin clot (Fig. 4 B/D).

The corneal tissue (epithelial and stromal fragments), which had been removed during lamellar keratoplasty, was processed for histological assessment. Histological analysis of the corneal epithelium showed thickening and slight keratinization of the epithelium with PAS-positive granular deposits in the superficial layers (Fig. 4 F). Bowman's layer was absent and the stromal layers were irregular with focal necrosis and few inflammatory cells around PAS-positive material (possibly non-vital fungal cells, Fig. 4 G).

## 4 Discussion

In this case series, we report the clinical, AS-OCT, and histological findings of four patients with keratomalacia in VAD. Serum vitamin A levels were reduced below detection level (<20 ng/ml) in two out of the four cases. The other two patients had subnormal vitamin A levels, but these patients had further risk factors (chronic posterior blepharitis and exposition keratopathy) for corneal ulceration. Typical Bitot's spots were found only in one of our cases (#1). Bitot's spots might be missing even in cases with severe VAD as in case #2. In one patient (#3), the cornea had been overgrown with conjunctival tissue, which showed keratinization (Fig. 3 C) and massive thickening of the epithelium detected by AS-OCT. Thickening of the epithelium of the cornea and conjunctiva was documented and followed by AS-OCT (e.g. Fig. 1 B/F, Fig. 3 D). Interestingly, none of our patients had severe pain. Instead they reported ocular irritation and reduced visual acuity as main symptoms. Although tested corneal sensitivity was normal or subnormal (Table 1), ocular perforation had not been noticed in these patients as a sudden or painful event. Three out of four patients perceived oligosymptomatic corneal perforation and required tectonic anterior lamellar keratoplasty. In case #3, we presume that a corneal perforation in the right eye had occurred in the past with spontaneous healing by conjunctivalization of the cornea and keratoplasty was not indicated in this advanced stage and poor visual prognosis. The main histological findings of the excised corneal tissue were massive epithelial thickening (keratinizing metaplasia), a pronounced granular layer of the corneal epithelium, and absence of substantial inflammatory reaction or infiltration by microorganisms (Fig. 1, Fig. 2, Fig. 4). In case #4, the histological specimen obtained during keratoplasty was free from microorganisms, showing that the infections had been successfully treated by antimycotics and antibiotics, respectively. Nevertheless, the cornea finally perforated despite the application of an amniotic membrane patch to treat the persistent epithelial wound.

In countries with sufficient general food supply, VAD with ocular manifestation is rare, but possibly underdiagnosed. Rubino et al. reported on three patients with VAD due to malnutrition for multiple food allergies, previous bowel bypass surgery, and Crohn's disease.^4^ In contrast to our case series, patients had only Bitot's spots, but no keratomalacia or corneal perforation. Consistent with the reports in the literature, perforation in patients with VAD can be oligo- or asymptomatic. Apart from the corneal sensitivity, reduced perception of corneal pain might also be explained by altered dopamine and serotonin metabolism in the brain, which has been found in patients with chronic alcohol consumption.^14^ Magno et al. reported, that increased alcohol consumption (>10 g/day) led to decreased perception of dry eye symptoms in male patients, although objective parameters for dry eye worsen in heavy drinkers.^14^^,^^15^ However, corneal perforation in VAD is also painless in non-alcoholics: Martin et al. reported the case of a woman with VAD due to primary biliary cirrhosis and secondary Sjögren's syndrome, who also suffered from painless bilateral corneal perforation.^8^ Our case # 3 denied regular or heavy alcohol consumption, but had not noticed the previous perforation of her right cornea either. There are only few recent reports about histological findings in corneas with VAD. For example, Khoramnia published the case of a corneal perforation in a 52 year-old patient with chronic alcoholism.^13^ Histologically, this enucleated eye showed corneal ulceration with infiltration of the cornea by lymphocytes and plasma cells, and acanthotic corneal epithelium. Sharp demarcation of necrotic stromal lamellae to normal stromal tissue without relevant inflammation is another histological hallmark of VAD.^16^ AS-OCT might help to objectively measure the corneal/conjunctival thickness in VAD and to monitor success of treatment in patients with vitamin supplementation for VAD. There are no completely unique or specific OCT signs in patients with VAD. The thickening of the conjunctiva in Bitot's spots in VAD is different from neoplastic lesions. In VAD, the epithelium itself is thickened leading to a hyperreflective layer in AS-OCT which forms folds and waves. On the contrary, neoplastic lesions of the conjunctiva (ocular surface squamous neoplasias) are also hyperreflective in AS-OCT but have a smooth or papillary surface and affect usually not the entire conjunctiva but only clearly demarcated areas. An abrupt transition from neoplastic hyperreflective epithelium to normal hyporeflective epithelium has been described.^17^ Overhanging edges are another sign for malignancy.^18^

Apart from the causative treatment strategy by vitamin A supplementation, ocular treatment in xerophthalmia consists of intensive application of lubricants and antibiotics in case of bacterial keratitis. Ointments containing vitamin A are especially helpful in this situation. Corneal perforation can be treated with tectonic keratoplasty, corneal gluing, or amniotic membrane transplantation.^5^^,^^7^^,^^10^ Wound healing in eyes requiring keratoplasty for corneal perforation in VAD is poor, leading to repeat keratoplasties or further interventions as amniotic membranes, botulinum toxin injections in the eyelid, or even evisceration.^7^^,^^8^ We usually perform anterior lamellar keratoplasties to cover perforated or deep stromal ulcers. The main advantage of this technique consists in the reduced risk of graft rejections since the patient's own endothelium is preserved. This is of advantage especially in patients with ocular surface disease as in VAD, in which topical steroids (necessary to avoid rejections) might disturb epithelial wound healing. The visual outcome of this technique might be inferior to perforating keratoplasties. However, the objective of keratoplasty in the event of a perforated corneal ulcer is tectonic rather than optic. If the ocular surface stabilizes over time, a secondary penetrating keratoplasty for visual rehabilitation is still possible. Therefore, we preferred tectonic lamellar anterior keratoplasty to penetrating keratoplasty, since the latter renders the additional risk of endothelial immune rejection.

The findings of our case series might support clinicians to gain better insight in the diagnosis and monitoring of patients with ocular manifestations of VAD: First, clinicians should be aware of the possibility of VAD as cause of corneal ulceration or perforation and should initiate laboratory analysis of serum vitamin A level, even if the typical Bitot's spots are not present. The true number of patients with VAD might be underestimated if the classical signs of xerophthalmia are missing, especially in countries with sufficient general food supply. Secondly, AS-OCT has been shown as useful tool to document the disturbance of the ocular surface in VAD and to measure the thickness of the conjunctiva and cornea. Furthermore, the very fast improvement of the corneal surface (as in case #1) within a few weeks after vitamin A supplementation can be monitored by AS-OCT. Thirdly, comparison of AS-OCT and histological findings indicated that AS-OCT imaging had falsely simulated that there was sufficient remaining stroma, but the bulging tissue seen clinically and in AS-OCT consisted histologically mainly of thickened epithelium which does not contribute to stromal stability. Thus, clinicians should be aware that thickness measurements in keratomalacia must be interpreted differently than in normal corneas. The study is limited by the small number of cases and the relatively short follow-up time. Furthermore, the case series was inhomogenous since two of the four patients had only subnormal vitamin A levels.

In conclusion, ocular signs of VAD are seen rarely in countries with sufficient general food supply, but can lead to severe corneal complications up to perforation. It is important to consider VAD as cause of delayed corneal wound healing or melting even if obvious corneal or conjunctival xerosis (Bitot's spots) are not always present. Chronic alcoholism and cachexia were the most common causes of VAD in our case series. AS-OCT can help to detect thickened corneal epithelium consistent with reduced epithelial turnover. Taking a detailed medical history and awareness of the possibility of VAD is mandatory in the treatment of patients with corneal ulcers.

## Patient consent

Consent to publish these case reports has been obtained from the patients in writing.

## Funding

No funding or grant support

## Authorship

All authors attest that they meet the current ICMJE criteria for authorship.

## CRediT authorship contribution statement

**Julia M. Weller:** Writing – original draft, Visualization, Validation, Methodology, Investigation, Formal analysis, Conceptualization. **Theofilos Tourtas:** Writing – review & editing, Validation, Supervision, Formal analysis, Data curation. **Friedrich E. Kruse:** Writing – review & editing, Validation, Supervision, Data curation.

## Declaration of competing interest

The following authors have no financial disclosures: JMW, TT, FEK.
